# Special Issue “Gynaecological Cancers Risk: Breast Cancer, Ovarian Cancer and Endometrial Cancer”

**DOI:** 10.3390/cancers14020319

**Published:** 2022-01-10

**Authors:** Ranjit Manchanda

**Affiliations:** 1 Wolfson Institute of Population Health, Queen Mary University of London, London EC1M 6BQ, UK; r.manchanda@qmul.ac.uk; 2 Department of Gynaecological Oncology, Barts Health NHS Trust, Whitechapel Road, London E1 1BB, UK; 3 Department of Health Services Research, Faculty of Public Health & Policy, London School of Hygiene & Tropical Medicine, London WC1E 7HT, UK

Over the last decade there have been significant advances and developments in our understanding of factors affecting women’s cancer risk, our ability to identify individuals at increased risk and risk stratify populations, as well as implement and evaluate strategies for screening and prevention. This special issue of *Cancers (Basel)*, through a series of 13 original articles and three reviews, captures some of the important advances in cancer risk, genetic testing, risk management, screening and prevention of breast, ovarian and endometrial cancers.

Our understanding of the genetic risk of ovarian cancer has significantly improved over the last decade. Pavanello et al. [1] provide an overview of the genetic landscape of ovarian cancer and summarise the evidence and estimates of various rare pathogenic variants (PVs) associated with an increased risk of ovarian cancer. Gaba et al. [2] provide pilot data from the first population-based testing implementation study, providing personalised ovarian cancer risk estimates to general population women. They demonstrate that this approach of personalised population-based OC risk stratification is feasible, acceptable, has high satisfaction, reduces cancer worry/risk perception and does not negatively impact psycho-social well-being or quality of life. This sets the stage for larger implementation studies to follow. In a randomised experimental survey of general population women, Gallagher et al. [3] show that women are willing to undergo risk reducing surgery to reduce their ovarian cancer risk at a range of risk levels and that uptake rates are similar for 5–10% and >10% life time ovarian cancer risks. For the first time, Manchanda et al. [4] demonstrate the cost-effectiveness of population-based *BRCA* testing across multiple high income and upper middle income countries health systems (USA, UK, Netherlands, China and Brazil). This strategy could prevent tens of thousands more breast and ovarian cancers than the current family history-based clinical approach. While this is potentially cost-saving for high income countries, genetic testing costs need to fall further for this to be cost-effective for low income countries. Kalsi et al. [5] show that an annual ultrasound-based screening strategy for ovarian cancer is not suitable as it misses 37.5% of cancers and does not downstage disease. Ovarian cancer screening using the Ca125 biomarker alone also has not demonstrated a mortality benefit [6]. Gentry-Maharaj et al. [7] evaluate the potential for using multi-marker longitudinal algorithms incorporating Ca125, HE4, CA72-4 and anti-TP53 autoantibodies for general population screening for ovarian cancer in post-menopausal women. However, none of the combinations improved the performance of using longitudinal Ca125 alone. Screening for ovarian cancer remains a conundrum which requires further research. Funston et al. [8] systematically evaluate various diagnostic tools used for early diagnosis of ovarian cancer in symptomatic women. Four tools with similar moderate accuracy are described and areas for further research are highlighted. Chandrasekaran et al. [9] demonstrate the importance of implementing parallel panel germline and somatic testing for women at ovarian cancer diagnosis. A panel-based approach increases the yield of PVs and parallel testing identifies large genomic rearrangements that would have otherwise been missed. Kondrashova et al. [10], using tumor signature analysis, highlighted a number of inheritable cancer susceptibility genes which may be associated with the development of endometrial cancer. Njoku et al. [11] provide initial evidence supporting a potential lipid biomarker-based strategy for endometrial cancer screening in women with elevated body mass index (BMI) who are at an increased risk of this disease. Alnafakh et al. [12] highlight that dyskerrin may be a regulator for endometrial cancer proliferation and a prognostic marker, opening up avenues for further research in this area. Dibden et al. [13] undertook a worldwide review and meta-analysis of cohort studies evaluating mammography-based breast cancer screening programmes, and found a 22% reduction in breast cancer mortality. Atakpa et al. [14] evaluated the association of weight loss (using diet and exercise) for breast cancer risk reduction with changes in breast density in pre-menopausal women. While short-term reduction in BMI is associated with a reduction in fatty breast tissue, it was not associated with changes in glandular or dense breast tissue, indicating that breast density may not capture any weight-loss associated reduction in breast cancer risk. Leventea et al. [15], using data from the PROCAS (Predicting Risk of Cancer at Screening) study, showed that while menopausal hormone therapy was associated with a higher risk of breast cancer, the risk is attenuated by an increase in BMI and adjusting for current BMI, the effect of hormonal therapy was not modified by early BMI or age of first pregnancy. Trebo et al. [16] establish that high Galectin-7 and low Galectin-8 expression are poor prognostic markers for breast cancer, highlighting the need for more research to comprehend the role of galectins in the regulation and interaction of tumor cells and macrophages. Howell et al. [17] describe risk assessment and management outcomes of one of the largest cohorts of women (14,311) seen in a tertiary-level high-risk service for women at increased risk of breast cancer.

GLOBOCAN data predict that breast, ovarian and endometrial cancer cases will increase by 47–53% and deaths by 58–71%, respectively, over the next 20 years [18]. A total of 70–90% of healthcare expenditure is directed at chronic disease management of which cancers is the second most common cause [19,20]. Improving primary and secondary prevention of cancers and other chronic diseases, will be critical for the future viability of our health systems. This issue makes an important contribution to the huge and swiftly advancing knowledge base across the area of ovarian, endometrial and breast cancer risk prediction, screening, prevention and personalised medicine. Greater funding and research efforts need to be directed towards screening and cancer prevention.

## References

[1] Pavanello M., Chan I.H., Ariff A., Pharoah P.D., Gayther S.A., Ramus S.J. (2020). Rare Germline Genetic Variants and the Risks of Epithelial Ovarian Cancer. Cancers.

[2] Gaba F., Blyuss O., Liu X., Goyal S., Lahoti N., Chandrasekaran D., Kurzer M., Kalsi J., Sanderson S., Lanceley A. (2020). Population Study of Ovarian Cancer Risk Prediction for Targeted Screening and Prevention. Cancers.

[3] Gallagher A., Waller J., Manchanda R., Jacobs I., Sanderson S. (2020). Women’s Intentions to Engage in Risk-Reducing Behaviours after Receiving Personal Ovarian Cancer Risk Information: An Experimental Survey Study. Cancers.

[4] Manchanda R., Sun L., Patel S., Evans O., Wilschut J., De Freitas Lopes A.C., Gaba F., Brentnall A., Duffy S., Cui B. (2020). Economic Evaluation of Population-Based BRCA1/BRCA2 Mutation Testing across Multiple Countries and Health Systems. Cancers.

[5] Kalsi J., Gentry-Maharaj A., Ryan A., Singh N., Burnell M., Massingham S., Apostolidou S., Sharma A., Williamson K., Seif M. (2021). Performance Characteristics of the Ultrasound Strategy during Incidence Screening in the UK Collaborative Trial of Ovarian Cancer Screening (UKCTOCS). Cancers.

[6] Menon U., Gentry-Maharaj A., Burnell M., Singh N., Ryan A., Karpinskyj C., Carlino G., Taylor J., Massingham S.K., Raikou M. (2021). Ovarian cancer population screening and mortality after long-term follow-up in the UK Collaborative Trial of Ovarian Cancer Screening (UKCTOCS): A randomised controlled trial. Lancet.

[7] Gentry-Maharaj A., Blyuss O., Ryan A., Burnell M., Karpinskyj C., Gunu R., Kalsi J.K., Dawnay A., Marino I.P., Manchanda R. (2020). Multi-Marker Longitudinal Algorithms Incorporating HE4 and CA125 in Ovarian Cancer Screening of Postmenopausal Women. Cancers.

[8] Funston G., Hardy V., Abel G., Crosbie E.J., Emery J., Hamilton W., Walter F.M. (2020). Identifying Ovarian Cancer in Symptomatic Women: A Systematic Review of Clinical Tools. Cancers.

[9] Chandrasekaran D., Sobocan M., Blyuss O., Miller R.E., Evans O., Crusz S.M., Mills-Baldock T., Sun L., Hammond R.F.L., Gaba F. (2021). Implementation of Multigene Germline and Parallel Somatic Genetic Testing in Epithelial Ovarian Cancer: SIGNPOST Study. Cancers.

[10] Kondrashova O., Shamsani J., O’Mara T.A., Newell F., McCart Reed A.E., Lakhani S.R., Kirk J., Pearson J.V., Waddell N., Spurdle A.B. (2021). Tumor Signature Analysis Implicates Hereditary Cancer Genes in Endometrial Cancer Development. Cancers.

[11] Njoku K., Campbell A.E., Geary B., MacKintosh M.L., Derbyshire A.E., Kitson S.J., Sivalingam V.N., Pierce A., Whetton A.D., Crosbie E.J. (2021). Metabolomic Biomarkers for the Detection of Obesity-Driven Endometrial Cancer. Cancers.

[12] Alnafakh R., Saretzki G., Midgley A., Flynn J., Kamal A.M., Dobson L., Natarajan P., Stringfellow H., Martin-Hirsch P., DeCruze S.B. (2021). Aberrant Dyskerin Expression Is Related to Proliferation and Poor Survival in Endometrial Cancer. Cancers.

[13] Dibden A., Offman J., Duffy S.W., Gabe R. (2020). Worldwide Review and Meta-Analysis of Cohort Studies Measuring the Effect of Mammography Screening Programmes on Incidence-Based Breast Cancer Mortality. Cancers.

[14] Atakpa E.C., Brentnall A.R., Astley S., Cuzick J., Evans D.G., Warren R.M.L., Howell A., Harvie M. (2021). The Relationship between Body Mass Index and Mammographic Density during a Premenopausal Weight Loss Intervention Study. Cancers.

[15] Leventea E., Harkness E.F., Brentnall A.R., Howell A., Evans D.G., Harvie M. (2021). Is Breast Cancer Risk Associated with Menopausal Hormone Therapy Modified by Current or Early Adulthood BMI or Age of First Pregnancy?. Cancers.

[16] Trebo A., Ditsch N., Kuhn C., Heidegger H.H., Zeder-Goess C., Kolben T., Czogalla B., Schmoeckel E., Mahner S., Jeschke U. (2020). High Galectin-7 and Low Galectin-8 Expression and the Combination of both are Negative Prognosticators for Breast Cancer Patients. Cancers.

[17] Howell A., Gandhi A., Howell S., Wilson M., Maxwell A., Astley S., Harvie M., Pegington M., Barr L., Baildam A. (2020). Long-Term Evaluation of Women Referred to a Breast Cancer Family History Clinic (Manchester UK 1987–2020). Cancers.

[18] International Agency for Research on Cancer (2018). Cancer Tomorrow. A Tool That Predicts the Future Cancer Incidence and Mortality Burden Worldwide from the Current Estimates in 2018 Up until 2040.

[19] Department of Health Long Term Conditions Team (2012). Long Term Conditions Compendium of Information.

[20] Murphy S.L., Xu J.Q., Kochanek K.D., Curtin S.C., Arias E. (2017). Deaths: Final Data for 2015. Natl. Vital Stat. Rep..

